# Supplementary figures and images for: Antifungal properties of volatile organic compounds produced by Daldinia eschscholtzii MFLUCC 19-0493 isolated from Barleria prionitis leaves against Colletotrichum acutatum and its post-harvest infections on strawberry fruits
Source: PeerJ. 2021 Apr 16;9:e11242. doi: 10.7717/peerj.11242 (PMC8054736; doi:10.7717/peerj.11242)

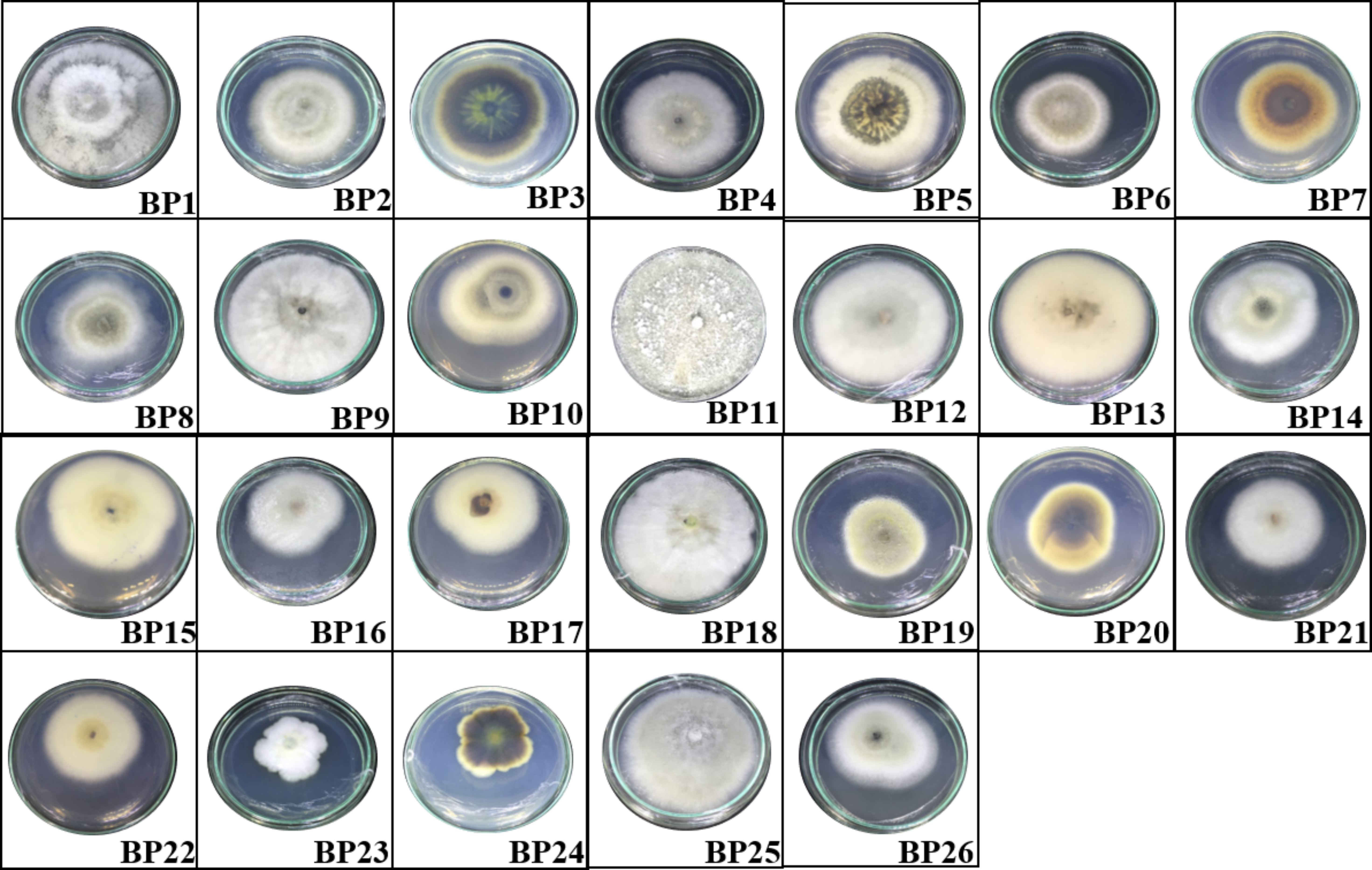

Supplement: Figure S1 — All endophytic fungi were cultured in PDA at room temperature for two weeks. [file peerj-09-11242-s002.png]

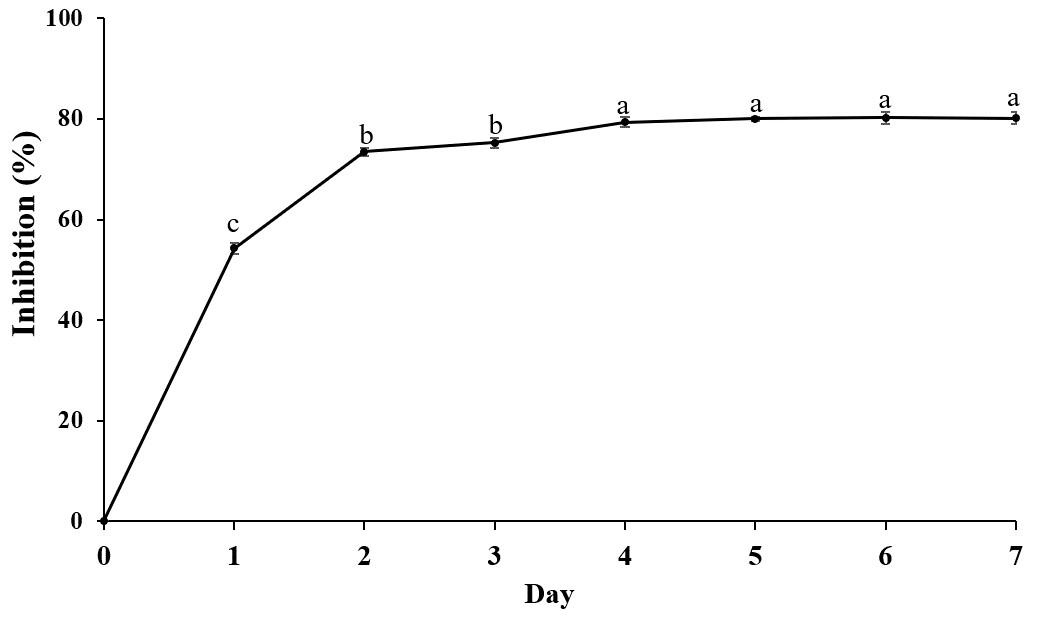

Supplement: Figure S2 — The data for each point are mean ± standard deviation (n = 15 per day). Different letters above the points indicate significant differences (p < 0.05) among the observed inhibition percentages across different days (ANOVA, followed by Duncan’s multiple range test). [file peerj-09-11242-s003.jpg]

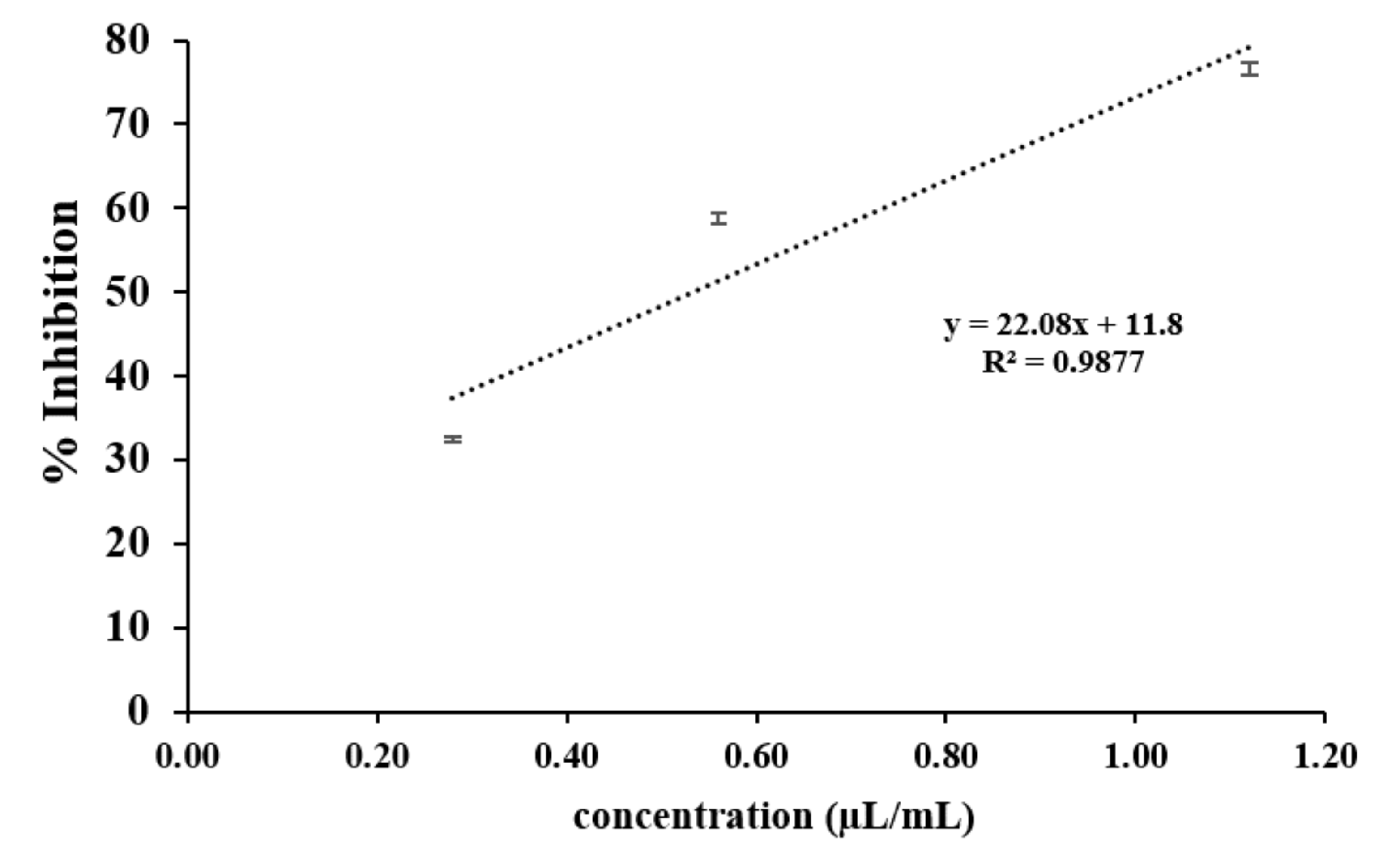

Supplement: Figure S3 — The data for each point are mean ± standard deviation (n = 15 for each concentration tested). [file peerj-09-11242-s004.png]
