# Supplementary material for: Antifungal properties of volatile organic compounds produced by Daldinia eschscholtzii MFLUCC 19-0493 isolated from Barleria prionitis leaves against Colletotrichum acutatum and its post-harvest infections on strawberry fruits
Source: PeerJ. 2021 Apr 16;9:e11242. doi: 10.7717/peerj.11242 (PMC8054736; doi:10.7717/peerj.11242)
Supplement: Table S1 — ATCC: American Type Culture Collection, Virginia, USA: CBS: Centraalbureau voor Schimmelcultures, Utrecht, The Netherlands, KC: Kew Culture Collection, United Kingdom, MFLUCC: Mae Fah Luang University Culture Collection, Chiang Rai, Thailand, MUCL: Université Catholique de Louvain, Belgium. [file peerj-09-11242-s005.docx]

**Table S1** Fungal species used in the phylogenetic analysis of *D. eschscholtzii* MFLUCC 19-0493 with the corresponding GenBank accession numbers.

| **Species** | **Strain number** | **GenBank accession numbers** | | |
| --- | --- | --- | --- | --- |
|  |  | **ITS** | **LSU** | **RPB2** |
| *A. atroroseum* | ATCC 76081 | AJ390397 | KY610422 | KY62423 |
| *A. michelianum* | CBS 119993 | KX376320 | KY610423 | KY624234 |
| *A. moriforme* | CBS 123579 | KX376321 | KY610425 | KY624289 |
| *A. nitens* | MFLUCC 12-0823 | KJ934991 | KJ934992 | KJ934994 |
| *A. stygium* | MUCL 54601 | KY610409 | KY610475 | KY624292 |
| *D. bambusicola* | CBS 122872 | KY610385 | KY610431 | KY624241 |
| *D. caldariorum* | MUCL 49211 | AM749934 | KY610433 | KY624242 |
| *D. dennisii* | CBS 114741 | JX658477 | KY610435 | KY624244 |
| *D. eschscholtzii* | KC 1616 | JX658496 |  |  |
| *D. eschscholtzii* | MFLUCC18-0177 | MK587659 | MK587746 | MK625010 |
| *D. eschscholtzii* | MUCL 47965 | JX658482 |  |  |
| *D. eschscholtzii* | CBS 117735 | JX658480 |  |  |
| *D. eschscholtzii* | MUCL 45434 | JX658484 |  |  |
| *D. eschscholtzii* | CBS 116037 | JX658492 |  |  |
| *D. eschscholtzii* | MUCL 43508 | JX658495 |  |  |
| *D. eschscholtzii* | CBS 113047 | AY616684 |  |  |
| *D. eschscholtzii* | CBS 116037(2) | JX658499 |  |  |
| *D. eschscholtzii* | CBS 116035 | JX658498 |  |  |
| *D. eschscholtzii* | MUCL 38740 | JX658493 |  |  |
| ***D. eschscholtzii*** | **MFLCCC 19-0493** | **MN704648** |  |  |
| *D. eschscholtzii* | CBS 117741 | JX658491 |  |  |
| *D. eschscholtzii* | MFLUCC19-0153 | MK587661 | MK587748 | MK625012 |
| *D. eschscholtzii* | CBS 113042 | JX658497 |  |  |
| *D. eschscholtzii* | CBS 116032 | JX658500 |  |  |
| *D. eschscholtzii* | MUCL 41777 | JX658486 |  |  |
| *D. eschscholtzii* | MUCL 41778 | JX658494 |  |  |
| *D. eschscholtzii* | KC1699 | JX658490 |  |  |
| *D. eschscholtzii* | CBS 117740 | JX658481 |  |  |
| *D. eschscholtzii* | CBS 122877 | JX658439 |  |  |
| *D. korfii* | STMA14089 | KY204020 |  |  |
| *D. loculatoides* | CBS 113279 | MH862918 | KY610438 | KY624247 |
| *D. macaronesica* | CBS 113040 | KY610477 | KY610477 | KY624294 |
| *D. petriniae* | MUCL 49214 |  | KY610439 | KY624248 |
| *D. placentiformis* | MUCL 47603 | AM749921 | KY610440 | KY624249 |
| *D. pyrenaica* | MUCL 53969 | KY610413 |  | KY624274 |
| *D. steglichii* | MUCL 43512 | KY610399 | KY610479 | KY624250 |
| *D. theissenii* | CBS 113044 | KY610388 | KY610441 | KY624251 |
| *D. vernicosa* | CBS 119316 | KY610395 | KY610442 | KY624252 |
| *E. liquescens* | ATCC 46302 | KY610389 | KY610443 | KY624253 |
| *H ticinense* | CBS 115271 | JQ009317 | KY610471 | KY624272 |
| *H. carneum* | MUCL 54177 | KY610400 | KY610480 | KY624297 |
| *H. cercidicola* | CBS 119009 | KC968908 | KY610444 | KY624254 |
| *H. crocopeplum* | CBS 119004 | KC968907 | KY610445 | KY624255 |
| *H. fendleri* | MUCL 54792 | KF234421 | KY610481 | KY624298 |
| *H. fragiforme* | MUCL 51264 | KC477229 | KM186295 | KM186296 |
| *H. fuscum* | CBS 113049 | KY610401 | KY610482 | KY624299 |
| *H. griseobrunneum* | CBS 331073 | KY610402 | KY610483 | KY624300 |
| *H. haematostroma* | MUCL 53301 | KC968911 | KY610484 | KY624301 |
| *H. howeanum* | MUCL 47599 | AM749928 | KY610448 | KY624258 |
| *H. hypomiltum* | MUCL 51845 | KY610403 | KY610449 | KY624302 |
| *H. investiens* | CBS 118183 | KC968925 | KY610450 | KY624259 |
| *H. lateripigmentum* | MUCL 53304 | KC968933 | KY610486 | KY624304 |
| *H. lenormandii* | CBS 119003 | KC968943 | KY610452 | KY624261 |
| *H. monticulosum* | MUCL 54604 | KY610404 | KY610487 | KY624305 |
| *H. musceum* | MUCL 53765 | KC968926 | KY610488 | KY624306 |
| *H. nicaraguense* | CBS 117739 | AM749922 |  |  |
| *H. papillatum* | ATCC 58729 | KC968919 | KY610454 | KY624223 |
| *H. perforatum* | CBS 115281 | KY610391 | KY610455 | KY624224 |
| *H. petriniae* | CBS 114746 | KY610405 | KY610491 | KY624279 |
| *H. porphyreum* | CBS 119022 | KC968921 | KY610456 | KY624225 |
| *H. pulicicidum* | CBS 122622 | JX183075 | KY610492 | KY624280 |
| *H. rubiginosum* | MUCL 52887 | KC477232 | KY610469 | KY624266 |
| *H. samuelsii* | MUCL 51843 | KC968916 | KY610466 | KY624269 |
| *H. submonticulosum* | CBS 115280 | KC968923 | KY610457 | KY624226 |
| *H. trugodes* | MUCL 54794 | KF234422 | KY610493 | KY624282 |
| *J. multiformis* | CBS 119016 | KC477234 | KY610473 | KY624290 |
| *R. angolense* | CBS 126414 | KY610420 | KY610459 | KY624228 |
| *R. pseudoannulata* | MUCL 51394 | KY610406 | KY610494 | KY624286 |
| *R. terebratum* | CBS 119137 | DQ631943 | DQ840069 | DQ631954 |
| *T. dendroidea* | CBS 123578 | FN428831 | KY610467 | KY624232 |
| *X. hypoxylon* | CBS 122620 | KY610407 | KY610495 | KY624231 |
| *X. polymorpha* | MUCL 49884 | KY610408 | KY610464 | KY624288 |

ATCC: American Type Culture Collection, Virginia, USA: CBS: Centraalbureau voor Schimmelcultures, Utrecht, The Netherlands, KC: Kew Culture Collection, United Kingdom, MFLUCC: Mae Fah Luang University Culture Collection, Chiang Rai, Thailand, MUCL: Université Catholique de Louvain, Belgium.
